# Schoolchildren with asymptomatic malaria are potential hotspot for malaria reservoir in Ethiopia: implications for malaria control and elimination efforts

**DOI:** 10.1186/s12936-023-04736-7

**Published:** 2023-10-16

**Authors:** Abdissa Biruksew, Ashenafi Demeke, Zewdie Birhanu, Lemu Golassa, Masrie Getnet, Delenasaw Yewhalaw

**Affiliations:** 1 School of Medical Laboratory Sciences, Faculty of Health Sciences, Institute of Health, Jimma University, Jimma, Ethiopia; 2 Tropical and Infectious Diseases Research Center (TIRC), Jimma University, Jimma, Ethiopia; 3 Arba Minch Health Science College, Arba Minch, Ethiopia; 4 Department of Health, Behavior, and Society, Faculty of Public Health, Institute of Health Jimma University, Jimma, Ethiopia; 5 Aklilu Lemma Institute of Pathobiology, Addis Ababa University, Addis Ababa, Ethiopia; 6 Department of Biostatistics and Epidemiology, Faculty of Public Health, Institute of Health, Jimma University, Jimma, Ethiopia

**Keywords:** Malaria prevalence, Asymptomatic malaria, Schoolchildren, Real-time PCR, Gomma district

## Abstract

**Background:**

Schoolchildren with asymptomatic malaria infections often go undiagnosed and untreated, serving as reservoirs for infection that hamper malaria control and elimination efforts. In this context, little is known about the magnitude of asymptomatic malaria infections in apparently healthy schoolchildren in Ethiopia. This study was aimed at determining the prevalence of asymptomatic malaria infection and its associated factors in apparently healthy schoolchildren in Ethiopia.

**Methods:**

From September 2021 to January 2022, a school-based cross-sectional study was conducted on 994 apparently healthy schoolchildren (aged 6–15 years) selected from 21 primary schools in the Gomma district, of Jimma zone, southwestern Oromia, Ethiopia. A multi-stage sampling technique was used to select schools and participants. After allocating the total sample proportionally to each school and then to each grade, participants were selected using the lottery method from a list of student records (rosters). Finger-pricked blood samples were collected for microscopy blood film preparation and malaria rapid diagnostic test (RDT) (SD Bioline Malaria Ag Pf/Pv). Moreover, dry blood spots (DBSs) were prepared onto filter papers for quantitative real time polymerase chain reaction (qPCR) analysis.

**Results:**

As determined by RDT and microscopy, the prevalence of asymptomatic malaria was 2.20% and 1.51%, respectively. Using qPCR, the overall prevalence was 5.03% (50/994). Of this, *Plasmodium falciparum*, *Plasmodium vivax* and mixed infections accounted for 90%, 6% and 4%, respectively. Submicroscopic asymptomatic malaria infection was also accounted for 70% (35/50) of the overall prevalence. Household head age, nighttime outdoor activities of household heads, family history of malaria, absence of insecticide-treated nets (ITN), and presence of stagnant water around the houses are all significantly associated with asymptomatic malaria infections among schoolchildren.

**Conclusions:**

This study found that both RDT and microscopy underestimated the prevalence of asymptomatic malaria in schoolchildren. However, qPCR was able to detect even low levels of parasitaemia and revealed a higher prevalence of asymptomatic submicroscopic malaria infections. The findings imply that schoolchildren with asymptomatic malaria infection are potential hotspot for malaria reservoir that fuels ongoing transmission. Therefore, it is imperative to include schoolchildren and schools in malaria intervention package and equally important is the adoption of more advanced and sensitive diagnostic tools, which would be crucial for successful malaria control and elimination efforts. Targeted interventions for asymptomatic malaria-infected schoolchildren can provide invaluable support to the National Malaria Control Programme in controlling and eventually eliminating the disease.

**Supplementary Information:**

The online version contains supplementary material available at 10.1186/s12936-023-04736-7.

## Background

Malaria, an ancient scourge that has plagued humanity for half a million years [1], holds a dark legacy as it is estimated to have claimed the lives of half of all people who have ever lived [2]. The causative agents of this disease are five different species of *Plasmodium* parasites (*Plasmodium falciparum, Plasmodium vivax, Plasmodium ovale, Plasmodium malariae* and *Plasmodium. knowlesi*), which are transmitted into the human body through the bite of female *Anopheles* mosquitoes, which seek a blood meal to sustain themselves [3].

Malaria can present in various forms, from severe and complicated cases to mild and uncomplicated instances to asymptomatic malaria [4]. Therefore, asymptomatic malaria, often referred to as "silent threat" [5], is a significant portion of the population where individuals are infected with the malaria parasites but do not exhibit any visible symptoms of the disease, and represents a reservoir for sustained transmission [6]. This makes them invisible to healthcare systems and serves as a source of persistent transmission [7, 8]. In this regard, the demography in sub-Saharan Africa is bottom-wide, with more than 27% of the population being young or of school age (5–15 years) [9], and nearly 40% of Ethiopians are under the age of 15 [10]. Hence, school-age children, the proportion of the population serving as malaria reservoirs, are more attractive and available to mosquito bites, contributing to the overall infectious reservoir for malaria [11], as they have received little attention and have benefited only marginally from the universal malaria intervention[12–14]. In this age group, repeated infections lead to the development of partial immunity against future complications, which aids in the establishment of asymptomatic reservoirs [15], and are reportedly more infectious than symptomatic patients [4]. In Uganda, for instance, a study conducted in areas with rigorous mosquito control measures and high *P. falciparum* transmission has revealed a startling finding: school-age children carrying asymptomatic malaria reservoirs were responsible for a staggering 50.4% of malaria transmission to mosquitoes [16]. In the same vein, Okell et al*.* [17] estimated that asymptomatic submicroscopic carriers (the proportion of malaria cases missed by microscopy) account for 20–50% of human-to-mosquito transmission. Furthermore, an experimental study conducted in low-transmission areas of Ethiopia found that both microscopic and submicroscopic asymptomatic malaria-infected individuals contributed 76.2% and 15.8%, respectively, of human-to-mosquito transmissions [18]. The infections are particularly prevalent in stable malaria transmission regions of sub-Saharan Africa, possibly due to naturally acquired immunity that develops over time from repeated infections [19–22].

Since 2004, Ethiopia has significantly reduced burden of malaria through deploying numerous health extension workers for extensive community interventions and putting control measures into place [23]. As a result, it has succeeded in reducing malaria incidence by 40% by 2021, as set forth in the Global Technical Strategy (GTS). In 2021, the country estimated to account for 1.7% of cases and 1.5% of deaths of malaria in 2021, making it a serious public health concern. Roughly, 75% of the landmass is malarious, and about 27.20% of the population lives in high-risk areas. Besides this, transmission in the country is highly seasonal and unstable [24–26]. As part of its country-owned, country-driven malaria elimination program, Ethiopia has adopted a National Malaria Elimination Roadmap, which was recently revised as the National Malaria Elimination Strategic Plan 2021–2025 [23, 26] with the vision of a malaria-free Ethiopia by 2030. To this end, conventional malaria diagnostic tools (microscopy and malaria RDT), vector control, case treatment, and single-dose primaquine as a transmission blocker are used in the framework of universal health coverage [26]. National malaria control programmes (NMPs) and the global research community have largely been focused on symptomatic (clinical) malaria, however, it appears to have neglected the contribution of asymptomatic malaria-infected individuals to the global disease burden, undermining the true prevalence of the disease [27].

In healthcare systems, conventional malaria diagnostic tools are usually used for screening of symptomatic and self-presented patients, but they are inherently incapable of detecting such low-density parasitaemias, allowing the disease to spread. For instance, the limit of detection (LoD) of malaria microscopy up to 100 parasites/µL by expert microscopist, missing on average between 20–50% of the cases [17] and that of the most malaria RDTs is 100–200 parasites µL) [28, 29]. Such chronic infections in schoolchildren not only pose a challenge to the elimination efforts, but they are also associated with long-term health consequences, such as school-absenteeism and cognitive impairment [12]. Addressing the burden in asymptomatic schoolchildren through targeted interventions offers two broad benefits. First, it can help achieve Sustainable Development Goals 3 and 4: promoting healthy living and well-being for people of all ages and quality education for all [13, 30]. Second, prioritizing malaria control in this large demographic can be used as a strategy to reduce transmission, since this segment of the population is vulnerable to malaria and a significant source of ongoing infection [31]. Generating evidence of the magnitude of asymptomatic malaria reservoirs in schoolchildren is therefore critical to informing the NMPs and justifying the inclusion of schools in malaria control packages. However, little is known about asymptomatic malaria infection and its associated factors in apparently healthy schoolchildren in the Gomma District of the Jimma Zone in southwestern Oromia, Ethiopia.

## Methods

### Details of study setting and design

In the earlier work [32], a thorough description of the study was provided. Additionally, a concise explanation of the background with some additional information pertinent to the current work is presented here. According to the Ethiopian Statistical Service, southwestern district office [33], there are a total of 300,266 population dwelling the Gomma district, of which 152,402 are males and 147,864 are females, with 20.24% semiarid urban areas and the majority are rural residents. The district is composed of 42 lower-level administrative villages (known as *Ganda* in Afaan Oromoo). The district is well-known for its extensive coffee growing including moist Afromontane forests with various tree species as its climax vegetation. Gomma district is therefore receives rain all year-round and is endemic to malaria (unpublished, Jimma Zone Health and agriculture Departments, unpublished data]. Furthermore, according to data obtained from the Ethiopian Meteorology Institute, Southwestern Oromia Meteorology Research Center [unpublished data, 2021], Gomma district received an annual temperature range from 25 to 28 °C, creating an ideal environment for mosquito breeding. As a seasonal disease, malaria has a peak immediately following the main rainy season and then gradually declines over the following months. There are only two *Plasmodium* parasite species endemic to Jimma zone in general: *P. falciparum* and *P*. *vivax,* with *P. falciparum* is the leading cause of malaria. Malaria prevention and control are active in the district, except that ITN distribution has been halted for four years (2018–2022) due to a decrease in malaria incidence in the area. As a result, some households were still using old ones. Based on the country's malaria stratification [23] system, Jimma zone, including the Gomma district, is classified as having low malaria transmission depending on its Annual Parasite Incidence (API).

### Sample size and sampling technique

As described in the previously published work [32], the sample size (n) for this study was determined using the single population proportion formula,

$$n = \frac{{z \left(\frac{\alpha }{2}\right)}^{2} p(1 - p)}{{d}^{2}}$$

which took into account z = 1.96 at the desired confidence interval (CI) of 95% along with a 2% margin of error (d), and the expected prevalence rate(p) of asymptomatic malaria infection among apparently healthy schoolchildren in Gomma district was 6.8% [22]. Using these parameters, the sample size was calculated to be 608. However, to account for potential non-response, a 10% non-response rate was added, resulting in a total sample size of 668. To account for possible variability in the multi-stage sampling technique, a design effect of 1.5 was applied. This adjustment increased the final sample size to 1002 schoolchildren. This study utilized a multi-stage sampling technique. The Gomma district was randomly selected as the primary sampling unit from a list of 21 districts in Jimma zone. From a list of 70 schools, 21 secondary sampling units were selected using lottery method. The sample size was then allocated proportionally to each primary school, grade, and section (within grade). Each school child was selected as the final sampling unit using the lottery method from the sampling frames (student rosters), stratified by age (6–15 years) and grade levels (1–4 and 5–8) according to methods described by Brooker et al. [34] and previous works [22, 35].

### Inclusion and exclusion criteria

Schoolchildren aged 6 to 15 years, whose family/guardian had consented to the study, who were also not taking anti-malarial medication and had an auxiliary body temperature of < 37.5 °C were included. Schoolchildren who were taking anti-malarial medication within one month of enrollment, had a fever at the time of data collection, or were over 15 years old were excluded from the study.

### Demographic data collection

Using a structured questionnaire, both demographic and malaria risk factor data were collected through face-to-face interviews with a child's head of household while observational data were recoded. The data were collected by trained data collectors.

### Blood film examinations

Finger-pricked blood was collected from each student, and both thick and thin blood smears were prepared on a frosted-ended microscopic slide. After air-drying, the smears were stained for 10 min with 10% buffer-diluted Giemsa and examined under the microscope with a 100X oil immersion objective. Following the standard protocol [36] and also described in the previous work [32], skilled malaria microscopists at Jimma University detected, identified *Plasmodium* species, and determined asexual parasitaemia after reading the slides. Any discrepancies were confirmed by a third experienced microscopist who was unaware of the first two microscopists' readings. In addition, the HemoCue® Hb 301 System (with curves product date: 2020.10.29 and expiry date: 2022.10.29; lot number: 2101570; HemoCue AB Kuvettgattan 1, SE-262 71, REF 111801, Ängelholm, Sweden) was used to measure the haemoglobin (Hb) concentration (in g/dL) from finger-pricked blood samples blood samples [37]. In accordance with WHO recommendations, anaemia (after Hb adjusted for altitude) is then categorized as having an Hb concentration of less than 11.5 g/dL in children aged 5 to 11 years and less than 12.0 g/dL in those aged 12 to 15 years [38].

### Malaria Rapid Diagnostic Test (RDT)

In this study, a malaria RDT, the SD Bioline Malaria Ag (Pf-HRP2 and Pv-pLDH) was used in the field to screen children for possible malaria infections. It is a three-banded device containing a *P. falciparum* HRP2, a *P. vivax* pLDH, and a control bands, manufactured by Standard Diagnostics Inc. (now Abbot Diagnostic Korea Inc.) with LOT number: 05DDF011A, and product code 05fk80, Korea. The kit was developed in order to detect histidine-rich protein-2 (pfHRP2) of *P. falciparum* and lactate dehydrogenase (pLDH) of *P. vivax*. As previously stated [32], after labelling the device with the students’ ID, 5µL of blood samples were collected for onsite testing, and the kit provided results in 15 min (per the manufacturer's instructions). It is commonly utilized in the country’s rural healthcare facilities (heath post and health centers) to rule out malaria in febrile individuals.

### Molecular assays

The same procedures described in the earlier publication of a section of this work was applied in this section as well [32]. Using a round hole puncher, approximately 3–5 mm diameter pieces of DBS were cut into a labelled 1.5 ml Eppendorf tube. The previously described Chelex-100 -resin- saponin method was then used to extract parasite DNA from DBS. The purified DNA was transferred to Nunc tubes with a volume of 0.5 mL and stored at -20 °C until it was ready for use in the PCR assay. The PCR assay was then conducted using QuantStudio™ 3 Real-Time Multiplex PCR. This molecular tool was used to amplify the 18S rRNA genes, allowing for the detection of both *P. falciparum* and *P. vivax*. The amplification process involved the use of a pair of forward and reverse primer sequences and probes that were specifically designed for this purpose. For *P. falciparum* specific primers (5’-3’) [39]: F-F (forward): TATTGCTTTTGAGAGGTTTTGTTACTTTG, and F-R (Revers): ACCTCTGACATCTGAATACGAATGC. And probe used was: Pf-fam (MGB): ACGGGTAGTCATGATTGAGTT. For *P. vivax* specific primers used was: Pv-1 (forward): CGCTTCTAGCTTAATCCACATAACTG, and Pv-2 (Reverse): AATTTACTCAAAGTAACAAGGACTTCCAAG. The probe used was: Pv-probe (VIC-MGB): CGCATTTTGCTATTATGT [40]. To ensure the utmost precision in the amplification process, we included positive controls consisting of DNA from *P. falciparum* and *P. vivax* isolates (Pf and Pv MR4(BEI) whereas molecular grade water was used as a negative control. For the qPCR master mix, the PerfeCTa® qPCR ToughMix® (Low ROX™, Quanta Bio vwr, catalog number: 97065–968) was utilized to guarantee optimal performance. The PCR amplification was conducted in a total reaction volume of 12 μL, comprising of 6 μL of the PerfeCTa master mix. To target *P. falciparum* and *P. vivax,* 0.5 μL (× 2) each of the Pf-Fam and Pv-vic probes, along with 0.4 μL (× 4) each of the forward and reverse primers were used. Furthermore, 2 μL of extracted DNA and 1.4 μL of molecular grade water were added. Under the specified PCR cycling conditions, which involved 45 cycles, the amplification process commenced with an initial denaturation at 50ºC for 2 min. Subsequently, 45 cycles of amplification were performed at 95ºC for 2 min, followed by 2 s at 95ºC and 30 s at 60ºC. The entire run-time for this process lasted approximately 60 min.

### Data management and statistical analysis

Data were coded, entered, and cleaned using EpiData version 3.1 and exported to SPSS version 26.0 for analysis. Descriptive and inferential statistics were done as frequencies and percentages to determined socio-demographic characteristics and prevalence of malaria among asymptomatic schoolchildren. A chi-square test was used to determine whether there was a statistically significant association between schoolchildren characteristics and asexual parasite density as measured by microscopy. Finally, both bivariate and multivariate logistic regressions model were used to assess factors associated with asymptomatic malaria infection in apparently healthy schoolchildren. Variables with a p-value of < 0.25 in the bivariate model were included in the final model. Variables with a p-value < 0.05 from the multivariate model were considered statistically significant association with the outcomes. An adjusted odds ratio with 95% confidence interval (CI) was used as the association measure. The goodness of fit of the model was assessed using the Hosmer–Lemeshow test.

### Ethical consideration

This project received ethical approval from the Institutional Review Board (IRB) (Ref: IHRPED/550/2) of the Institute of Health at Jimma University. The study was also approved by the Jimma zone Health and Education offices, and official letters were submitted to the Gomma district Health and Education offices, then to each school director. Furthermore, final permission was obtained from each schoolchild's household head.

## Results

### Characteristics of the study participants

Beforehand, 1002 healthy schoolchildren were enrolled in the study; however, eight were excluded based on exclusion criteria, which gave a response rate of 99.20%. Thus, a total of 994 healthy children with normal auxiliary temperature (< 37.5 °C) were included for this analysis. Of the study participants, 52.40% (521/994) were males and 47.60% (473/994) were females. The mean age of the study participants was 11.54 years (± 2.5 SD). Over half (56.40%) of the children were in their 1^st^ cycle primary education (grade 1–4) while 43.60% were in their 2^nd^ cycle primary education (grades 5 – 8). The majority of schoolchildren (77%) were from rural areas and 23% from semi-urban areas. The locations of study sites lie at an altitude between 1428 and 1749 m above sea level (masl) with a mean altitude of 1623 masl (Table 1).

**Table 1:** Socio-demographic characteristics of the study participants in Gomma District, Jimma Zone, Southwestern Oromia, Ethiopia, 2022

Characteristics of participants	Category	n (%)
Socio-demographic characteristics		
Student’s sex	Male	521 (52.40)
	Female	473 (47.60)
Student’s age group	6–11	463 (46.6)
	12–15	531 (53.40)
Student’s grade level	1–4	561 (56.40)
	5–8	433 (43.60)
Sex of the household head (HHH)	Male	915 (92.10)
	Female	79 (7.90)
Age of the HHH	< 35	72 (7.20)
	35–44	295 (29.70)
	45–54	374 (37.60)
	≥ 55	253 (25.50)
Marital status of the HHH	Married	911 (91.60)
	Widowed	49 (4.90)
	Divorced	30 (3.00)
	Single	4 (0.40)
Educational status of the HHH	Cannot read and write	326 (32.80)
	1–8	544 (54.70)
	9–12	49 (4.90)
	Tertiary level	75 (7.50)
	Farmer	794 (79.90)
Occupation of the HHH	Gov’t employee	94 (9.50)
	Merchant	76 (7.60)
	Daily labor	30 (3.00)
Household size	< 5	442 (44.50)
	≥ 5	552 (55.50)
Residency of the household	Rural	765 (77)
	Semi-urban	229 (23)

n = number

### Prevalence of asymptomatic malaria reservoirs

**Fig. 1:**
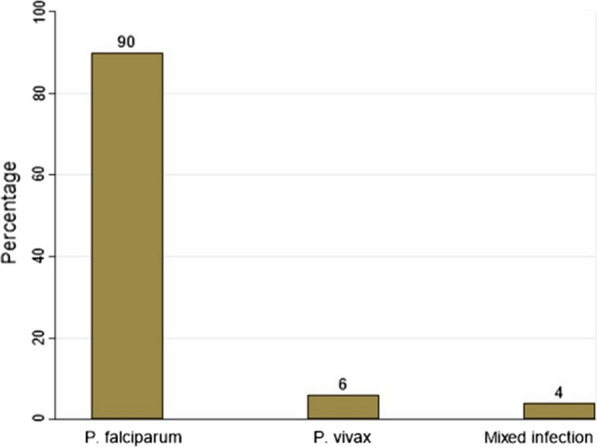
Prevalence of *Plasmodium* species as determined by qPCR in asymptomatic malaria infected school children in Gomma zone, Oromia, Ethiopia

Of the 994 healthy school children from 21 primary schools included in the study (Additional file 1: Tables A and B), using RDT, the prevalence of asymptomatic malaria was 2.20% (95% CI: 1.40, 3.30) whereas using microscopy, it was 1.51% (95% CI: 0.80, 2.50), However, only *P. falciparum* mono infections were detected with both conventional malaria diagnostic tools. The overall prevalence determined by qPCR (Table 4) was 5.03% (95% CI: 3.80, 6.60). Of these, 6.33% (33/50) were males and 3.60% (17/50) were females. The majority [80% (40/50)] of children were from rural while and 20% (10/50) were from semi-urban areas. There was no difference in the proportion of children infected in either age group (6–11 years or 12–15 years), with each group accounting for 50% (25/50). The prevalence in grades 1–4 was 54% (27/50), while it was 46% (23/50) in grades 5–8. *Plasmodium falciparum, P. vivax*, and mixed infections accounted for 90% (45/50), 6% (3/50), and 4% (2/50) of all infections, respectively. Of *P. falciparum* infected schoolchildren, 64.44% (29/45) was males whereas 35.56% (16/45) was females (Fig. 1).

Furthermore, Hb concentration was measured to determined level of anaemia, which ranged between 10.5 and 14.80 g/dl, with a mean of 12.62 g/dl (± 0.82 SD). As a result, children with moderate anaemia (< 11.5 g/dl) accounted for 5.84% (n = 58), of whom 32.76% (n = 19) were infected with malaria. Of these malaria-infected, mildly anaemic children, 78.95% (n = 15) were infected with *P. falciparum*, 15.80% (n = 3) with *P. vivax*, and a mixed infection. On the other hand, the proportion of children with normal Hb levels (> 11.5 g/dl) was 94.16% (n = 936), of which 31 (3.31%) were asymptomatic malaria carriers. The majority of these infections (n = 30) were caused by *P. falciparum* (n = 30), with one mixed infection.

*Plasmodium falciparum* asexual parasite densities were determined in all microscopically positive children, and ranged from 64 to 8080 parasites/L (Table 2). Furthermore, it was observed that more than half of these children were gametocyte carriers, infective stage of *Plasmodium* species to female *Anopheles* mosquito. As demonstrated in Table 2, there was no statistically significant association between parasite density and schoolchildren's characteristics.

**Table 2:** Distribution of asexual stages of *P. falciparum* density by sex, age group, grade level, place of residence and altitude in asymptomatic malaria infected schoolchildren in Gomma district

Characteristics		Parasite density/μL					
		< 200	201–500	501–2000	2001–5000	5001–9000	p-value
Sex	Female	2	1	0	0	0	0.504
	Male	3	2	3	3	1	
Age (in years)	6–11	2	1	2	1	1	0.710
	12–15	3	2	1	2	0	
Grade level	1–4	2	1	2	2	1	0.710
	5–8	3	2	1	1	0	
Residence	Semi-urban	2	0	0	0	0	0.329
	Rural	3	3	3	3	1	
Altitude (masl)	< 1600	2	1	3	1	0	0.306
	≥ 1600	3	2	0	2	1	

Statistically significant when p-value of the Chi square (X^2^) test is < 0.05

### Proportions of malaria cases missed by microscopy and malaria RDT

In this study, the proportion of cases missed by microscopy is translated to asymptomatic submicroscopic malaria infection, which is obtained by subtracting the microscopic prevalence from the qPCR prevalence (50–15 = 35). This means that 35 children went un diagnosis and left un treatment. (Table 3). As a result, the prevalence of submicroscopic asymptomatic malaria was 70% (35/50), with *P. falciparum* made up 85.70% (30/35), followed by *P. vivax* [8.60% (3/35)] and mixed infections [5.70% (2/35)]. Although the RDT detected seven-fold more cases than microscopy, it also missed 56% (28/50) of infection reservoirs detected by qPCR. Generally, both the conventional malaria diagnostic tools missed a high number of infection reservoirs, which might jeopardize malaria elimination efforts.

**Table 3:** Proportions of asymptomatic malaria infections missed by conventional malaria diagnostic tools in the total qPCR prevalence in asymptomatic malaria infected schoolchildren in Gomma district

		+ ve	-ve	Total	% missed
Microscopy	+ ve	15	0	15	$\frac{35}{50}*100=70\%$
	− ve	35	944	979	
Total		50	944	994	
RDT	+ ve	22	0	22	$\frac{28}{50}*100=56\%$
	−ve	28	944	972	
Total		50	944	994	

### Factors associated with asymptomatic malaria infection in schoolchildren

Bivariate and multivariate logistic regressions were employed to identify factors associated with asymptomatic malaria among schoolchildren. As the summary of results can be seen in Table 4, the analysis identified six variables that are significantly (P < 0.05) associated with an increased risk of malaria infection in asymptomatic schoolchildren: anemic schoolchildren, household head’s (HHH) age, HHH nighttime outdoor activities, family history of malaria, household ITN ownership, and presence of stagnant water near the household.

**Table 4:** Bivariate and multivariate analysis of factors associated with asymptomatic malaria in schoolchildren in Gomma district, Jimma Zone, Oromia, Ethiopia

Individual child factors	Categories	All	n (%)		COR (95% CI)	p-value	AOR (95% CI)	p- value
			+ve	−ve				
Sex	Female	473	17 (3.60)	456 (96.40)	1		1	
	Male	521	33 (6.33)	488 (93.66)	1.814 (0.99, 3.30)	0.051		
Age (in years)	6–11	463	25 (5.40)	438 (94.6)	1.16 (0.65, 2.04)	0.621		
	12–15	531	25 (4.70)	506 (95.30)	1			
Grade level	1–4	561	27 (4.81)	534 (95.19)	0.90 (0.51, 1.59)	0.72		
	5–8	433	23 (5.30)	410 (94.70)	1			
Slept inside ITN the previous	No	74	10 (13.50)	64 (86.50)	0.589 (0.212, 1.64)	0.31		
	Yes	83	7 (8.43)	76 (91.57)	1			
Previous history of malaria	No	942	42 (4.58)	900 (95.42)	1			
	Yes	52	8 (15.38)	44 (84.62)	3.89 (1.72, 8.79)	0.001		
Hb level(g/dl)	< 11.5	58	19 (32.76)	39 (67.24)	14.22 (7.38, 27.37)	< 0.0001	12.05 (5.329, 27.274)	< 0.0001***
	≥ 11.5	936	31 (3.31)	905 (96.69)	1		1	
Household factors								
Sex of the household head (HHH)	Female	79	1 (1.27)	78 (98.73)	1			
	Male	915	49 (5.35)	866 (94.65)	4.41 (0.60, 32.40)	0.14		
Age of the HHH	< 35	72	3 (4.17)	69 (95.83)	1			
	35–44	295	11 (3.73)	284 (96.27)	0.89 (0.24, 3.28)	0.86		
	45–54	374	13 (3.50)	361 (96.50)	0.83 (0.23, 2.98)	0.77		
	≥ 55	253	23 (9.10)	230 (90.90)	2.30 (0.67, 7.89)	0.18	4.98 (1.218, 20.645)	0.027*
Educational status of HHH	Cannot read and write	326	16 (4.90)	31 (0 95.10)	0.723 (0.256, 2.038)	0.54		
	1–8	544	25 (4.60)	519 (96.40)	0.674 (0.250,01.819)	0.44		
	9–12	49	4 (8.20)	45 (91.80)	1.244 (01.819, 1.819	0.75		
	Tertiary	75	5 (6.70)	70 (93.80)	1			
Night-time outdoor activities of HHH	No	643	21 (3.27)	622 (96.73)	1		1	
	Yes	351	29 (8.26)	332 (91.74)	2.67(1.50, 4.75)	0.001	3.27 (1.602, 6.687)	0.001**
Family history of malaria infection	No	742	49 (6.60)	693 (93.40)	1		1	
	Yes	252	1 (0.40)	252 (99.4)	0.056 (.008, 0.410)	0.005	0.02 (0.003, 0.159)	< 0.0001**
Household ITN ownership	No	837	33 (3.94)	804 (96.06)	2.958 (1.604, 5.456)	0.001	3.62 (1.676, 7.838)	0.001**
	Yes	157	17 (10.83)	140 (89.17)	1			
Environmental factors								
Presence of stagnant water around home	No	446	9 (2.00)	437 (98.00)	1		1	
	Yes	548	41 (7.50)	507 (92.50)	3.93(1.89, 8.17)	0.0001	4.98 (2.042, 12.178)	< 0.0001**
Place of residency	Rural	765	40 (5.23)	725 (94.77)	0.83 (0.41, 1.68)	0.60		
	Semi-urban	229	10 (4.37)	219 (95.63)	1		1	
Altitude of the house (masl)	< 1600	312	20 (6.41)	292 (93.59)	1.489 (0.831, 1.266)	0.18		
	≥ 1600	682	30 (4.40)	652 (95.60)	1			

*COR* Crude Odds ratio, *AOR* Adjusted odds ratio

*Significant at p < 0.05, **significant at p < 0.01, ***Significant at p < 0.001

Accordingly, apparently healthy anemic schoolchildren were 12 times more likely to be asymptomatic malaria carriers than non-anaemic children [AOR = 12.05 (95% CI: 5.329, 27.274)]. Also, children whose HHHs were 55 years or older were five times more likely to be infected with malaria than children whose HHHs were younger than 35 years [AOR = 4.98 (95% CI: 1.218, 20.645)]. In the same vein, children whose HHHs engaged in nighttime outdoor activities had a 3.27 [AOR = 3.27 (95% CI: 1.602, 6.687)] times greater risk of developing asymptomatic malaria compared to whose heads of households did not engage in such activities. What stands out in the Table 4 is that children with a family history of malaria were 98% less likely to be infected with malaria infection than children without a family history of malaria [AOR = 0.021 (95% CI: 0.003, 0.159)]. The study found that there were 3.6 times higher likelihood of malaria infection in children living with a family who did not have ITNs compared to children with a family who owned ITN [AOR = 3.624 (95% CI: 1.676, 7.838)]. Notably, children who lived in homes near stagnant water were 4.65 times highly likely [AOR = 4.986 (95% CI: 2.042, 12.178)] contracting malaria than children who lived in homes without nearby stagnant water.

## Discussion

Asymptomatic malaria infections pose a hidden threat to malaria elimination efforts. This is because large segments of the population, asymptomatic schoolchildren with malaria, are sources of persistent infection and contribute to continued transmission [8, 12, 34, 41]. To ensure the success of the malaria elimination program, it is critical to address this segment of the population, which did not receive special attention in the malaria control and elimination strategy [8, 12] and the burden of infections in this age group remains unaddressed across endemic countries [30]. As a result, the current study investigated the prevalence of asymptomatic malaria infection and its associated factors in healthy schoolchildren in the Gomma district of Jimma zone, Southwestern Oromia, Ethiopia.

In this study, the overall prevalence of asymptomatic malaria infection was 5.03%, as determined by qPCR. The majority of infections were caused by *P. falciparum* (90%) followed by *P. vivax* (6%), and mixed infections (4%). The prevalence of falciparum malaria infection was 1.51% using RDT and 2.20% using microscopy. This is consistent with other reports [23, 25], according to which *P. falciparum* infections account for the vast majority of infections in sub-Saharan countries. Importantly, 70% of the schoolchildren in this study were asymptomatic submicroscopic malaria reservoirs. The majority of infections (85.70%) were caused by *P. falciparum*, followed by *P. vivax* (8.60%) and mixed infections (5.70%), highlighting that microscopic asymptomatic infection is only the tip of the iceberg, consistent with previous findings in Ethiopia [42]. The result of this study is in line with those of Okell et al*.* [17], that looked at data from a number of different studies that had been conducted in areas with low malaria transmission. They also found that in areas with a microscopy prevalence of < 10%, the prevalence of submicroscopic infections could range from 70 to 80%. As previously described [17], the approach to calculating the asymptomatic submicroscopic malaria prevalence was as follows:

$${\text{Submicroscopic prevalence}} = {\text{qPCR prevalence}} - {\text{Microscopic prevalence}}.$$

Whitttaker et al. [7] also conducted systematic review and meta-analysis showed that asymptomatic submicroscopic infections are more common in low malaria transmission areas. In this study, the RDT also missed a significant proportion (56%) of infections detected by qPCR.

In broad sense, the current study showed that males had a higher prevalence of asymptomatic malaria than females, which is consistent with previous research from Ghana [43]. Despite what might be expected, infections are typically similar regardless of age, grade level, place of residence or altitude of the household. A large body of evidence [8, 17, 44–46] suggests that such hidden infection are important sources of sustained transmission, accounting for 20–50% of all human-mosquito transmissions [17, 46–48] and *P. falciparum* infection demonstrates that it can remain in the circulation for months, up to 200 days [49]. The findings in this study imply that infections were actively and covertly spread among the study population, going undiagnosed as a result absence of health seeking behavior at healthcare systems. A handful of scientific studies explores the potential causes of these infections: From the immunological viewpoint, older children develop asymptomatic states as a result of intermittent, non-sterilizing immunity following repeated exposure, leading to low-density patatitaemias [50]. Because of this, once infection is established, both anti-parasite and anti-disease immune reactions are triggered, and the parasite reacts in accordance with its priority. In low-transmission areas with few mosquito bites, the parasite opts to be low-parasitaemic and increase transmission at the cost of decreased pathogenicity in order to thwart an anti-parasitic immune response. However, because mosquito bites are common in high-transmission areas, the parasite favours overriding host immunity over transmission [51–53]. Another state of knowledge [54, 55] deals with the virulence trade-off hypothesis. According to this theory, parasites are typically less virulent in low-transmission settings to avoid competition from more virulent strains. They consequently persist at low densities and go undiagnosed, making them a transmission candidate. Therefore, less virulent strains would prevail over more virulent ones, with the latter being cleared after diagnosis thanks to anti-malarial treatment and a stronger immune response. The most recent discovery also revealed the fourth *Plasmodium* parasite life cycle, particularly *P. vivax*, in the human spleen [56, 57]. The scientists conducted an imperial study on splenectomized Indonesian patients who also had chronic, asymptomatic malaria infections. The PCR results revealed that 98.7% of *P. vivax* and 93.1% of *P. falciparum* hidden biomasses were found in the human spleen rather than in the circulation. It is therefore safe to conclude that malaria parasites escape detection by conventional methods by circumventing circulation in chronically asymptomatic carriers and evolving a novel survival strategy. Additionally, Andrade et al*.* [52] showed that longer *P. falciparum* circulating time is linked to increased splenic clearance in asymptomatic carriers during the dry season, and maintains parasitaemia below clinical and immunological thresholds. A similar study in Uganda reported that in areas where malaria is effectively controlled, asymptomatic school-age children were sources of persistent infections [8].

The microscopic result of this study is lower than reports from northern Ethiopia [18, 22, 58], Uganda [16], Democratic Republic of Congo, DRC [59], Cameroon [60], Ghana [61, 62], Tanzania [63] and India [64]. The variations could be attributed to different malaria transmission settings, study periods, and host immunity. In addition, since 2015, Ethiopia made significant strides in drastically reducing the incidence of malaria through the comprehensive implementation of control strategies and the implementation of the elimination programme. In contrast, this result is higher compared to previous study conducted in Ethiopia [42, 65]. In this study, the RDT-based result is comparable to a recent finding by Zerdo et al. [45] in asymptomatic schoolchildren in southern Ethiopia and lower than that of in the DRC [59], Kenya [66], Ghana [61] Similarly, the PCR result in this study is lower than the study reported in Ethiopia [42, 65], Uganda [8, 67] Ghana [44, 68], DRC [59], Cameron [60], Tanzania [63] and India [64]. However, it is higher compared to a study conducted by Tadesse et al*.*[69] on school-age children in Ethiopia. These differences appear to be due to differences in host immunities, parasite densities, and differences in studies conducted in different malaria transmission settings.

Furthermore, the present study identified several risk factors for asymptomatic malaria infection in schoolchildren, which may help malaria control programmes focus on targeted interventions. Proceeding sequentially, anemic schoolchildren were at a significantly higher risk of having asymptomatic malaria infection, which is consistent with reports from previous studies [43, 70, 71]. A possible explanation for this might be that an increased risk of anemia can lead to a transition from asymptomatic to severe malaria. Anaemia weakens the immune system, makes it harder for the body to fight off malaria parasites, and leaves the children more susceptible to serious infections. Similarly, children whose household heads were older than 55 years had a higher risk of developing asymptomatic malaria. As a result of the lack of access to diagnosis and treatment, malaria in children is influenced by the ages of the household heads [72]. Additionally, adults are more likely to be exposed mosquito bite [73] as older people tend to spend more evening hours active, unprotected by ITNs and occasionally engaged in nighttime outdoor activities, increasing the likelihood of exposing children to malaria-transmitting mosquito bites [74, 75]. One striking result of this study is that children who had a family history of malaria were 98% less likely to be infected with malaria infection than those with no history. This is mainly due to the fact that children who have a family history of malaria are more likely to have been exposed to the disease in the past, this exposure helps them build up their acquired immunity and makes them less likely to contract the disease [76]. In addition, once experiencing the disease, families would likely to take preventive measures to protect their children. It was noticed that schoolchildren living in households without ITN were more likely to contract malaria than those living in households with ITN, consistent with a previous study [43, 58]. The use of ITN is one of the effective ways to fight against malaria; however, studies show that this age group uses bed nets less frequently than the rest population [41] and benefits the least from universal malaria interventions [12]. It should be noted that according to the Gomma district Malaria Control department (unpublished data), the distribution of ITNs had been suspended prior to the start of this study (2018–2021) and the study presents here only 157 households using an older ITN. It's interesting to note that children whose families had not used IRS in the previous 3–12 months had a higher risk of having asymptomatic malaria than those whose families had used the IRS in that time. Likewise, school children who lived in a house where there was standing water nearby were at odds of the infection than those who did not live in such houses. This is supported by a study conducted in northwest Ethiopia [77, 78]. It could be due to the fact that such water bodies provide ideal mosquito breeding grounds, increasing the risk of children being bitten by mosquitoes carrying the malaria parasite [79]. However, this finding contradicts with a systematic review and meta-analysis of asymptomatic malaria in Ethiopia, which found no statistically significant association between stagnant water and asymptomatic malaria infection [80]. In contrast to earlier reports [43, 81, 82], this study found no statistically significant association between schoolchildren's sex and asymptomatic malaria infection. However, it seems that male children had more infections than female children. This disparity in malaria infections could be attributed to gender roles related to labor division and hormonal influence [83]. In experimental studies, male mice developed testosterone-linked susceptibility to and estrogen-linked resistance to malaria after infecting malaria-free mice [84, 85], as well as immunological differences [86], genetic factors, behavioral factors, and sleeping patterns [75]. This study, however, has certain limitations that should be taken into consideration. While this study provides valuable insights into the association between predictor variables and asymptomatic malaria infection schoolchildren, it is important to note that it is a cross-sectional study. This means that the data on anaemia and malaria were collected simultaneously, making it impossible to ascertain a cause-effect relationship between the two conditions. Therefore, readers should interpret these findings with caution.

## Conclusion

The current study revealed that apparently healthy schoolchildren were potential hotspot malaria infection reservoirs. The study also showed that infection was more common in male children than females and that both RDT and microscopy missed a significant portion of infection which tested positive with qPCR. The study also uncovers a multitude of factors associated with increased risks of apparently healthy schoolchildren to asymptomatic malaria. Hence, it was revealed that anaemia in children, the age of household heads, their nighttime outdoor activities, and the presence of stagnant water around housed all play pivotal roles in this phenomenon. Moreover, the study highlights the significance of a family history of malaria and the absence of ITNs as crucial contributors to this alarming issue. By shedding light on these interconnected factors, this study provides invaluable insights into the intricate web of connections associated with the increased risks of asymptomatic malaria in schoolchildren.

Inclusion of schoolchildren and schools in the malaria intervention package would be the most effective strategy for the success of malaria control and elimination programme. Given that asymptomatic malaria infected schoolchildren are the source of persistent infections, targeted intervention in conjunction with identified risk factors provides an additional opportunity to strengthen the National Malaria Control Programme. Hence, to ensure the elimination goal, such new interventions are strongly recommended to support and improve existing measures and it has the potential to provide both immediate and long-term benefits to schoolchildren. The authors also strongly recommend using molecular tools to screen for low-density parasitaemias in the malaria elimination programme.

### Supplementary Information

**Additional file 1: Table A**. Schools with number of asymptomatic malaria positive students in Gomma district, Jimma zone, Southwestern Oromia; from September 2021 to January 2022, Ethiopia. **Table B**. Study schools with their respective numbers of students participated in the study in Gomma district, Jimma zone, Southwestern Oromia; from September 2021 to January 2022, Ethiopia.

## Data Availability

The datasets used in the current study are available from the corresponding author upon request.
