# Supplementary material for: Schoolchildren with asymptomatic malaria are potential hotspot for malaria reservoir in Ethiopia: implications for malaria control and elimination efforts
Source: Malar J. 2023 Oct 16;22:311. doi: 10.1186/s12936-023-04736-7 (PMC10580533; doi:10.1186/s12936-023-04736-7)
Supplement: Supplementary file 1 — Additional file 1: Table A. Schools with number of asymptomatic malaria positive students in Gomma district, Jimma zone, Southwestern Oromia; from September 2021 to January 2022, Ethiopia. Table B. Study schools with their respective numbers of students participated in the study in Gomma district, Jimma zone, Southwestern Oromia; from September 2021 to January 2022, Ethiopia. [file 12936_2023_4736_MOESM1_ESM.docx]

# Additional file 1

**Table A**: Schools with number of asymptomatic malaria positive students in Gomma district, Jimma zone, Southwestern Oromia; from September 2021 to January 2022, Ethiopia.

| *Plasmodium* species +ve by qPCR | | | | | | | |
| --- | --- | --- | --- | --- | --- | --- | --- |
| School | | *P. falciparum* | *P. vivax* | Mixed infection | | Total | |
|  | Agamsa | 1 | 0 | | 0 | | 1 |
|  | Balto | 1 | 2 | | 0 | | 3 |
|  | Bulbulo | 0 | 1 | | 0 | | 1 |
|  | CederoSuse | 4 | 0 | | 0 | | 4 |
|  | Coocee | 8 | 0 | | 0 | | 8 |
|  | Dhidhess | 3 | 0 | | 0 | | 3 |
|  | GogaKamisee | 13 | 0 | | 1 | | 14 |
|  | GogaKilole | 3 | 0 | | 0 | | 3 |
|  | LimuShaye | 4 | 0 | | 1 | | 5 |
|  | Meexxii | 1 | 0 | | 0 | | 1 |
|  | Qoccolle | 3 | 0 | | 0 | | 3 |
|  | Qombaa | 4 | 0 | | 0 | | 4 |
| Total | | 45 | 3 | 2 | | 50 | |
| ***Plasmodium* species +ve by RDT** | | | | | | | |
| School | | P. falciparum | *P. vivax* | | Total | | |
|  | Agamsa | 1 | 0 | | 1 | | |
|  | Balto | 1 | 0 | | 1 | | |
|  | Cederosuse | 4 | 0 | | 4 | | |
|  | Coocee | 4 | 0 | | 4 | | |
|  | Dhidhessaa | 3 | 0 | | 3 | | |
|  | GogaKam | 3 | 0 | | 3 | | |
|  | GogaKilole | 2 | 0 | | 2 | | |
|  | Qoccolle | 3 | 0 | | 3 | | |
|  | Qombaa | 1 | 0 | | 1 | | |
| Total | | 22 |  | | 0 | | |
| ***Plasmodium* species +ve by microscopy** | | | | | | | |
| School | | P. *falciparum* | *P. vivax* | | Total | | |
|  | Agamsa | 1 | 0 | | 1 | | |
|  | Cederosue | 3 | 0 | | 3 | | |
|  | Coocee | 1 | 0 | | 1 | | |
|  | Dhidhessa | 1 | 0 | | 1 | | |
|  | GogaKamise | 3 | 0 | | 3 | | |
|  | GogaKillle | 2 | 0 | | 2 | | |
|  | Qoccolle | 3 | 0 | | 3 | | |
|  | Qombaa | 1 | 0 | | 1 | | |
| Total | | 15 |  | | 15 | | |

**Table B**. Study schools with their respective numbers of students participated in the study in Gomma district, Jimma zone, Southwestern Oromia; from September 2021 to January 2022, Ethiopia

| **Name of school** | | **No. students selected** | | **Percent** | |  |
| --- | --- | --- | --- | --- | --- | --- |
|  | Agamsa | | 16 | | 1.6 | |
|  | Andode | | 38 | | 3.8 | |
|  | Balto | | 53 | | 5.3 | |
|  | Bulbulo | | 52 | | 5.2 | |
|  | BurqaBeekumsa | | 19 | | 1.9 | |
|  | CederoSuse | | 39 | | 3.9 | |
|  | Coocee | | 69 | | 6.9 | |
|  | DedoUrache | | 19 | | 1.9 | |
|  | DhayiQacane | | 65 | | 6.5 | |
|  | Dhidhessaa | | 51 | | 5.1 | |
|  | Dinuu | | 38 | | 3.8 | |
|  | Ganjii | | 14 | | 1.4 | |
|  | Geembee | | 117 | | 11.8 | |
|  | GogaKamisee | | 58 | | 5.8 | |
|  | GogaKilole | | 55 | | 5.5 | |
|  | LimuShaye | | 87 | | 8.8 | |
|  | MeexxiiKoticha | | 19 | | 1.9 | |
|  | Qoccolle | | 33 | | 3.3 | |
|  | Qombaa | | 34 | | 3.4 | |
|  | Sadacha | | 37 | | 3.7 | |
|  | Yaacii | | 81 | | 8.1 | |
|  | Total | | 994 | | 100.0 | |
